# Alcohol-based decontamination of gloved hands: A randomized controlled trial

**DOI:** 10.1017/ice.2023.243

**Published:** 2024-04

**Authors:** Kerri A. Thom, Clare Rock, Gwen L. Robinson, Heather R.S. Reisinger, Jure Baloh, Emily Chasco, Yuanyuan Liang, Shanshan Li, Daniel J. Diekema, Loreen A. Herwaldt, J. Kristie Johnson, Anthony D. Harris, Eli N. Perencevich

**Affiliations:** 1 Department of Epidemiology and Public Health, University of Maryland School of Medicine, Baltimore, Maryland; 2 Division of Infectious Diseases, Department of Medicine, Johns Hopkins University, Baltimore, Maryland; 3 University of Iowa Carver College of Medicine, Iowa City, Iowa; 4 Department of Health Policy and Management, University of Arkansas for Medical Sciences, Little Rock, Arkansas; 5 MassMutual Data Science, Springfield, Massachusetts

## Abstract

**Objective:**

The gold standard for hand hygiene (HH) while wearing gloves requires removing gloves, performing HH, and donning new gloves between WHO moments. The novel strategy of applying alcohol-based hand rub (ABHR) directly to gloved hands might be effective and efficient.

**Design:**

A mixed-method, multicenter, 3-arm, randomized trial.

**Setting:**

Adult and pediatric medical-surgical, intermediate, and intensive care units at 4 hospitals.

**Participants:**

Healthcare personnel (HCP).

**Interventions:**

HCP were randomized to 3 groups: ABHR applied directly to gloved hands, the current standard, or usual care.

**Methods:**

Gloved hands were sampled via direct imprint. Gold-standard and usual-care arms were compared with the ABHR intervention.

**Results:**

Bacteria were identified on gloved hands after 432 (67.4%) of 641 observations in the gold-standard arm versus 548 (82.8%) of 662 observations in the intervention arm (*P* < .01). HH required a mean of 14 seconds in the intervention and a mean of 28.7 seconds in the gold-standard arm (*P* < .01). Bacteria were identified on gloved hands after 133 (98.5%) of 135 observations in the usual-care arm versus 173 (76.6%) of 226 observations in the intervention arm (*P* < .01). Of 331 gloves tested 6 (1.8%) were found to have microperforations; all were identified in the intervention arm [6 (2.9%) of 205].

**Conclusions:**

Compared with usual care, contamination of gloved hands was significantly reduced by applying ABHR directly to gloved hands but statistically higher than the gold standard. Given time savings and microbiological benefit over usual care and lack of feasibility of adhering to the gold standard, the Centers for Disease Control and Prevention and the World Health Organization should consider advising HCP to decontaminate gloved hands with ABHR when HH moments arise during single-patient encounters.

**Trial Registration:** NCT03445676.

The hands (and gloved hands) of healthcare personnel (HCP) contribute significantly to the spread of pathogens in the healthcare setting, and hand hygiene remains the cornerstone of prevention.^1^ Although much attention is given to hand hygiene at entry and exit to a patient room, there are many more opportunities (tasks/moments) for hand hygiene at the bedside as outlined by the World Health Organization (WHO) Five Moments campaign.^2^ Yet, adherence to the Five Moments is low, with reported compliance ranging from 22% to 60%,^3–6^ likely due to a high number of opportunities and insufficient time,^7–9^ which have been linked to noncompliance.^10^ Current recommendations state that HCP should remove gloves, perform hand hygiene, and don new gloves when a hand hygiene opportunity arises.^11,12^ Novel strategies to mitigate the burden of hand hygiene at the bedside, including reducing the time needed for each opportunity for hand hygiene, may in turn promote increased compliance and may ultimately be most effective in limiting transmission of infectious pathogens.^10,13,14^

In this study, we tested the novel strategy of using alcohol-based hand rub (ABHR) to decontaminate gloved hands (ie, the intervention) against the current recommendation of removing gloves, performing hand hygiene and donning new gloves (ie, the gold standard) and also against usual HCP care, which typically has poor compliance.^7,13,14^ We hypothesized that using an ABHR to directly decontaminate gloved hands when a hand hygiene opportunity arises during a single patient encounter would essentially be as effective as the current gold-standard recommendation to remove gloves, perform hand hygiene, and don new gloves before the next care task. Furthermore, we think that directly decontaminating gloved hands with ABHR would be superior to usual care by HCP given the low reported rates of adherence to the WHO Five Moments of Hand Hygiene. We tested this hypothesis using a randomized, 3-arm, intervention trial.

## Methods

**Figure 1.**
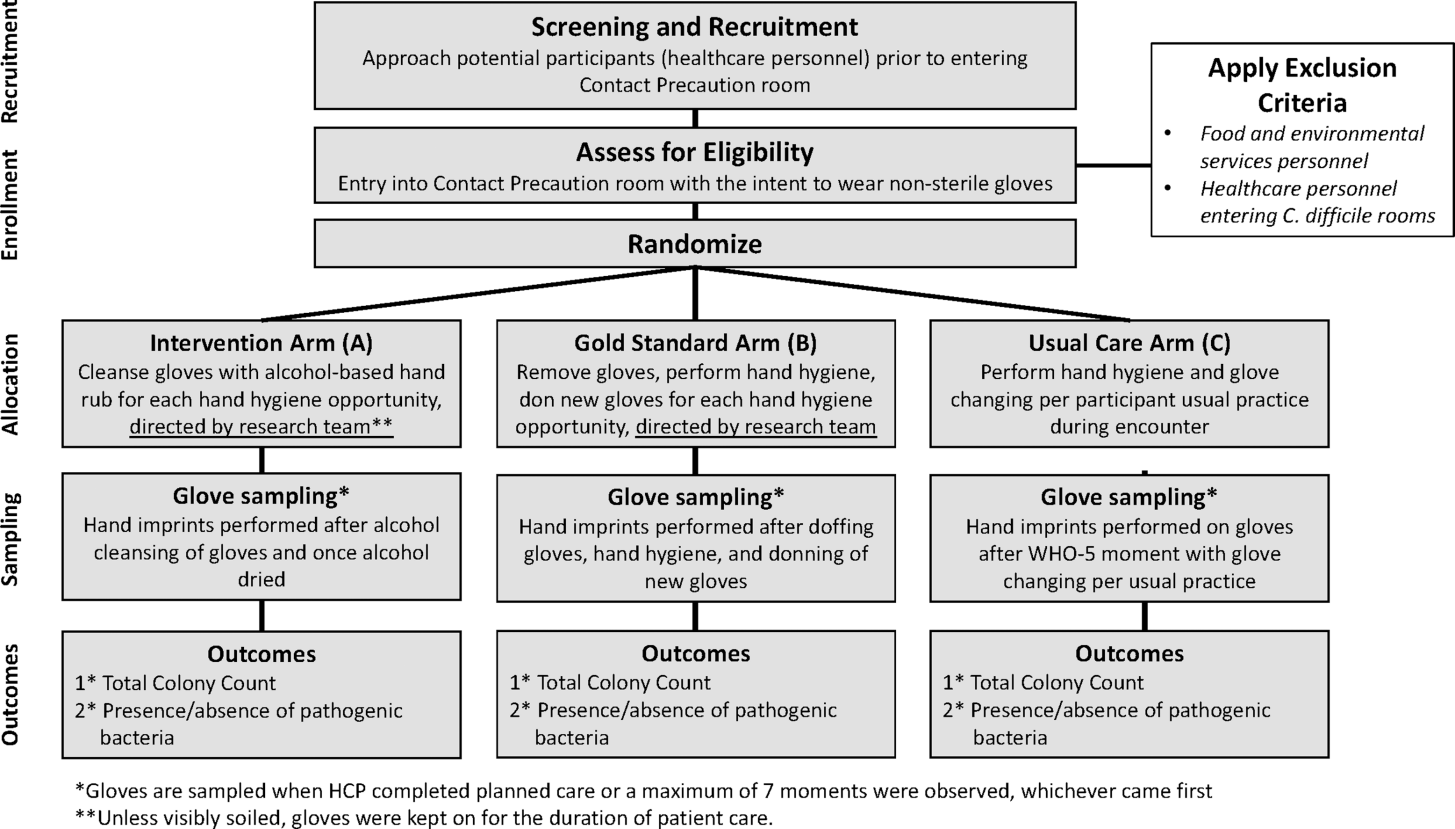
Study design.

We performed a mixed-method, multicenter, 3-arm, randomized trial to evaluate the efficacy of directly applying ABHR to decontaminate gloved hands compared with (1) the current gold standard and recommendation of glove removal, hand hygiene, and donning new gloves when an opportunity for hand hygiene arises at the bedside and (2) usual care. We compared the bacterial bioburden of gloved hands, as assessed by total colony count and detection of pathogenic bacteria, across the 3 study arms. The study design is illustrated in Figure 1.

From March 2017 to December 2018, we conducted this research across multiple clinical settings, including adult and pediatric medical-surgical units, intermediate care units, and intensive care units at 4 academic healthcare centers: The University of Maryland Medical Center, the R. Adams Cowley Shock Trauma Center, and the Johns Hopkins Hospital in Baltimore, Maryland and the University of Iowa Hospitals in Iowa City, Iowa. The University of Maryland–Baltimore Institutional Review Board (IRB) served as the central IRB, and this study was approved by central and local IRBs prior to data collection. This trial was conducted in accordance with the CONSORT statement.^15^

Participants were HCP who entered hospital rooms of patients on contact precautions with the intent to perform patient care. HCP entering rooms of patients on contact precautions for *Clostridioides difficile* were excluded from the study because soap and water, and not an ABHR, was standard hand hygiene practice during the care of these patients across the study sites. Dietary and environmental services personnel were also excluded because hand hygiene expectations for them differed from that expected of other HCP.

After providing verbal consent and before room entry, HCP were randomized into 1 of 3 study arms: intervention, gold standard, or usual care. We used a stratified, block-randomized scheme to ensure an equal number of participants from participating units in each arm. We instructed HCP to perform hand hygiene and don personal protective equipment at room entry as expected according to each site’s hospital infection prevention practice. Research coordinators used a modified WHO hand hygiene observation tool when observing HCP practice for all 3 study arms.

In the intervention arm, care activities were observed, and HCP were instructed by the research coordinator to use ABHR on gloved hands at each hand hygiene opportunity according to the WHO Five Moments of Hand Hygiene.^2^ In the intervention arm, gloves were not removed after each hand hygiene opportunity unless visibly soiled. HCP in the gold-standard arm were instructed by the research coordinator to remove gloves, perform hand hygiene, and don new gloves after drying at each hand hygiene opportunity. In the usual-care arm, HCP were observed but not given specific instruction to perform hand hygiene; behavior (ie, hand hygiene and/or glove change) was recorded for each opportunity. Healthcare workers were only randomized once because once randomized to an arm, they would be aware of the study and would be biased if enrolled again in another arm. For example, once in the intervention or gold-standard arm, their knowledge about the study would affect their subsequent behavior in the usual-care arm.

Research coordinators instructed participants in the intervention and gold-standard arms to perform hand hygiene with ABHR in a standardized manner^16^ and educated them on the WHO Five Moments of Hand Hygiene^2^ before they entered patients’ rooms so that they could identify hand hygiene opportunities. HCP randomized to the usual-care group received similar education after they participated in the study. Study participation ended when the HCP completed the planned patient care or had reached 7 hand hygiene opportunities, whichever came first. Participants who did not have at least 1 hand hygiene opportunity during the observation were not included in the final analysis. At the end of study participation (the end of the planned patient care or 7 hand hygiene opportunities), we used the direct imprint method to sampled HCP gloved hands as done in prior studies.^17^ In brief, we instructed participants to gently press the nondominant palm, thumb, and fingers directly onto the agar for 5 seconds each. We sampled the gloved hands of participants in the gold-standard arm after they performed hand hygiene and donned new gloves. We sampled the gloved hands of participants in the intervention arm after they performed hand hygiene on gloved hands and the alcohol had dried. We sampled the gloved hands of participants in the usual-care arm right after a WHO Moment as they moved to the next task. We gave participants the opportunity to change gloves if they intended to do so.

### Microbiologic and laboratory assessment

Participants hands were placed on trypticase soy agar with 5% sheep blood, 150 mm and the plates were incubated overnight at 35–37°C, then colony-forming units (CFUs) were counted. Potential pathogenic bacteria, Enterobacterales, *Staphylococcus aureus*, *Enterococcus* spp, *Pseudomonas aeruginosa*, and *Acinetobacter* spp were identified by first subculturing unique colonies onto trypticase soy agar with 5% sheep’s blood, MacConkey, and phenylethyl alcohol agar (Becton Dickenson, Sparks, MD). They were then worked up and identified using the Vitek or MALDI-TOF system.

We collected the gloves of participants after we sampled them, and we discarded gloves that were visibly soiled. We used a standardized approach to test the gloves for microperforations.^18^ We carefully turned gloves right-side out to avoid introducing new holes. We then poured water into each glove, stopping 1.27 cm (0.5 inches) below the top of the stretched glove. After 2 minutes, we inspected the outside surface of the glove for water accumulation.

### Power calculations

Power calculations were performed separately for the comparison of the intervention arm versus the gold-standard arm and for the intervention arm versus the usual-care arm. We performed this procedure because we expected a larger difference in effect size between the intervention arm versus usual-care arm than the intervention arm versus gold-standard arm. The power calculations estimated that 662 observations were needed in the intervention arm versus gold-standard arm to achieve 80% power and that 240 observations were needed in the intervention arm versus the usual-care arm.

### Statistical analysis

The gold-standard and usual-care arms were each independently compared with the intervention arm. A priori we determined that the outcome of total colony-forming units would be assessed both as categorical (high >30 CFU vs low ≤30 CFU)^17^ and continuous variables (mean CFU) based on a prior study. The proportion of high bacterial burden (>30 CFU) was compared between the 2 study arms using a χ^2^ test. The detection of pathogenic bacteria was similarly compared. Median CFUs were compared using the Mann-Whitney *U* test.

### Qualitative assessment

To assess potential facilitators and barriers to the use of ABHR to cleanse gloved hands during a single patient encounter, we conducted a qualitative evaluation of HCP perceptions of the intervention. We conducted semistructured interviews with a purposive sample of study participants across the 3 study arms (8–9 per arm), study sites, and groups of healthcare personnel including providers, nurses, nursing assistants, and allied health professionals. The team previously developed a codebook and qualitative methods a for a similar study^19^ that we used for the current qualitative analyses. Briefly, a qualitative analyst developed a code book consisting of inductive and deductive themes, thematically analyzed the data, extracted exemplar quotes, and conducted a theme frequency analysis.^20^

## Results

### Intervention versus gold standard

We observed 641 HCP–patient interactions in the gold-standard arm and compared the study outcomes in this group to 662 interactions in the intervention arm. Table 1 outlines the HCP and patient interaction characteristics in the 2 study groups. The mean time spent performing hand hygiene (ie, time taken to walk to the ABHR dispenser, apply ABHR to their gloved hands and resume patient care) was 14 seconds in the intervention arm. The mean time spent performing hand hygiene (ie, time taken to walk to the ABHR dispenser, remove their gloves, perform hand hygiene, allow hands to dry, put on new gloves and resume patient care) was 28.7 seconds in the gold-standard arm (*P* < .001). Bacteria were identified on the gloved hands of HCP after 980 (75.2%) of 1,303 observations: 432 (67.4%) of 641 observations in the gold-standard arm versus 548 (82.8%) of 662 observations in the intervention arm (*P* < .001). Total bacterial colony counts were lower in the gold-standard arm compared with the intervention arm: a median of 2 CFU (IQR, 0–5) versus 4 CFU (IQR, 1–15; *P* < .001). Potential pathogenic bacteria (as defined above) were identified on 25 (3.9%) 641 cultures in the gold-standard arm versus 48 (7.3%) 662 cultures in the intervention arm (*P* < .01).

**Table 1.** Gold-Standard Arm Versus Intervention Arm: Characteristics of the Observed Healthcare Personnel (HCP)–Patient Interaction and Study Outcomes

Variable	Ideal Standard (N = 641), No. (%)^a^	Intervention (N = 662), No. (%)^a^	Tota (N = 1,303), No. (%)^a^	*P* Value
**Healthcare personnel type**				.4^b^
Registered nurse	450 (70.2)	462 (69.8)	912 (70)	
Physician	40 (6.2)	29 (4.4)	69 (5.3)	
Allied health professional	58 (9.1)	69 (10.4)	127 (9.8)	
Other	92 (14.4)	102 (15.4)	194 (14.9)	
Unknown	1 (0.2)	…	1 (0.1)	
HCP right-hand dominance	581 (90.6)	598 (90.3)	1179 (90.5)	.96^b^
Morning observation vs afternoon/evening	515 (80.3)	513 (77.5)	1028 (78.9)	.23^b^
Time observed, mean minutes (SD)	9.0 (5.7)	8.2 (5.2)	8.6 (5.4)	.01^c^
**Hand hygiene opportunities observed**				.24^c^
Range	1–7	1–7	1–7	
Mean (SD)	3.1 (1.3)	3.2 (1.4)	3.2 (1.3)	
Median (IQR)	3 (2–4)	3 (2–4)	3 (2–4)	
Time spent doing hand hygiene, mean seconds (SD)^d^	28.7 (10.4)	14.0 (7.0)	21.0 (11.4)	<.0001
Micro-perforation	2/618 (0.3)	20/616 (3.0)	22/1234 (1.7)	<.001^b^
**Bacterial burden, colony counts**				
Gloves positive for bacteria	432 (67.4)	548 (82.8)	980 (75.2)	<.001^c^
Total, mean (SD)	7.4 (23.5)	21.4 (52.8)	14.5 (41.7)	<.001^e^
>30 CFU	24 (3.7)	87 (13.1)	111 (8.5)	<.001^e^
**Pathogen identified**	25 (3.9)	48 (7.3)	73 (5.6)	.01^e^
Gram-positive	10 (37.0)	15 (25.4)	25(29.1)	
Gram-negative	17 (63.0)	44 (74.6)	61(70.9)	

Note. CFU, colony-forming units; IQR, interquartile range; SD, standard deviation.

a Units unless otherwise specified.

b Fisher exact test.

c Mann-Whitney *U* test.

d Intervention arm: time taken to pump the alcohol on the gloves until they are finished rubbing the alcohol on their gloved hands and resume patient care). Gold-standard arm: time taken to remove the gloves, perform hand hygiene, all hands to dry, put on new gloves and resume patient care.

e χ^2^ test.

### Intervention versus usual care

We observed 135 HCP–patient interactions in the usual-care arm, study outcomes for this group were compared with the first 226 participants in the intervention arm. These 226 participants are a random subset of the 662 HCPs above (Table 2). In total, we recorded 537 opportunities for hand hygiene according to the WHO Five Moments in the usual-care arm (Table 3). HCP performed hand hygiene in only 11 (2%) of the observed opportunities, and they changed gloves in lieu of hand hygiene at an additional 15 opportunities (2.8%). Compliance was highest for moment 3, after contact with a patient’s blood or body fluid; HCP performed hand hygiene in 2 (4.7%) of 43 moments and changed gloves in 7 (16.3%) of 43 moments. Compliance was lowest for moment 5 (after contact with patient surroundings) and moment 1 (before patient contact).

**Table 2.** Usual Care Versus Intervention: Characteristics of the Observed Healthcare Personnel (HCP)–Patient Interaction and Study Outcomes

Variable	Usual Care (N = 135), No. (%)^a^	Intervention (N = 226), No. (%)^a^	Total (N = 361), No. (%)^a^	*P* Value
**Healthcare Personnel Type**				.71^b^
Registered nurse	99 (73.3)	174 (77.0)	273 (75.6)	
Physician	2 (1.5)	5 (2.2)	7 (1.9)	
Allied health professional	9 (6.7)	10 (4.4)	19 (5.3)	
Other	25 (18.5)	37 (16.4)	62 (17.2)	
Unknown	0 (0)	0 (0)	0 (0)	
Healthcare Personnel right hand dominance	125 (92.6)	209 (92.5)	334 (92.5)	
Morning observation vs afternoon/evening	98 (72.6)	165 (73.0)	263 (72.9)	1^c^
**Hand hygiene opportunities observed**				.07^c^
Range	1–7	1–7	1–7	
Mean (SD)	4.0 (1.7)	1.6 (1.5)	3.8 (1.6)	
Median (IQR)	4 (2–5)	4 (2–5)	4 (2–5)	
Time observed, mean minutes (SD)	9.3 (6.5)	9.3(5.2)	9.3 (5.7)	.33^d^
Microperforation	0/127 (0)	6/204 (2.9)	6/331 (1.8)	.14^b^
**Bacterial burden, colony counts**				
Gloves positive for bacteria	133 (98.5)	173 (76.6)	306 (84.8)	<.001^b^
Total, mean (SD)	84.1 (110.0)	23.5 (60.1)	46.2 (87.3)	<.001^d^
>30 CFU	62 (45.9)	34 (15.0)	96 (26.6)	<.001^c^
**Pathogen identified**	38 (28.1)	16 (7.1)	54 (15.0)	<.001^c^
Gram-positive	19 (43.2)	6 (30.0)	25 (39.1)	
Gram-negative	25 (56.8)	14 (70.0)	39 (60.1)	

Note. CFU, colony-forming units; IQR, interquartile range; SD, standard deviation.

a Units unless otherwise specified.

b Fisher exact test.

c χ^2^ test.

d Mann-Whitney *U* test.

**Table 3.** Opportunities for Hand Hygiene (HH) According to the WHO Five Moments and Healthcare Personnel Adherence Among Usual-Care Participants (N = 135)

	Total, No. (%)	Before Patient Contact (Moment 1), No. (%)	Before a Clean Procedure (Moment 2), No. (%)^a^	After Body Fluid Contact (Moment 3), No. (%)	After Patient Contact (Moment 4), No. (%)	After Contact with Surroundings (Moment 5), No. (%)
No. of opportunities^b^	537	168	106	43	207	208
Performed hand hygiene	11 (2)	1 (0.6)	3 (2.8)	2 (4.7)	6 (2.9)	2 (1)
Changed gloves	15 (2.8)	2 (1.2)	2 (1.9)	7 (16.3)	7 (3.4)	0
Performed hand hygiene or changed gloves	26 (4.8)	3 (1.8)	5 (4.7)	9 (20.9)	13 (6.3)	2 (1)

a Clean procedures included: device insertion (n = 1), wound dressing (n = 1), suctioning (n = 7), oral examination (n = 0), preparation of sterile materials (n = 8), opening circuit of device (n = 40), phlebotomy/injection (n = 22), bedside surgery (n = 0), preparation of medication (n = 23) and other (n = 3).

b An opportunity may have been counted for up to 2 WHO moments when transitioning between care activities; for example, if the healthcare personnel completed a physical exam (WHO moment ‘after patient contact’) and then performed a wound dressing (WHO moment ‘before a clean procedure’) the opportunity is counted in both categories.

Bacteria were identified on gloved hands of HCP after 306 (84.8%) of 361 observations: after 199 (98.5%) of 135 observations in the usual-care arm and 173 (76.6%) of 226 observations in the intervention arm (*P* < .001) (Table 2). Total bacterial colony counts were higher in the usual-care arm (median, 29 CFU; IQR, 10.5–105.5) compared with the intervention arm (median, 2 CFU; IQR, 1–14.75; *P* < .001). Potential pathogenic bacteria (as defined above) were identified on HCP hands after 38 (28.1%) of 135 observations in the usual-care arm versus 16 (7.1%) of 226 in the intervention arm (*P* < .001). Of the 331 gloves tested, 6 (1.8%) were found to have microperforations; no microperforations were found in the usual-care arm (0 of 127), and 6 (2.9%) of 204 gloves tested in the intervention arm had microperforations (*P* = .14).

### Qualitative findings: Perceived benefits and concerns

We interviewed 26 HCP, including 9 nurses, 8 physicians, 7 allied health professionals (eg, physical therapists and respiratory therapists), and 2 nursing assistants. Exemplar quotes for the themes of perceived benefits and concerns are shown in Table 4. When asked about perceived benefits of using ABHR to cleanse gloved hands, 21 (80%) of 26 perceived potential benefits: 14 perceived increased efficiency in time and effort, 5 perceived cost savings, 4 perceived less skin irritation, and 4 perceived extra protection. Also, 2 perceived improved mindfulness when performing tasks, 1 perceived improved guideline compliance, and 1perceived improved staff satisfaction. Also, 14 (54%) of 26 participants did not perceive any risks or concerns of using ABHR on gloved hands, as long as it was proven safe (ie, noninferior). The most cited concern was longer drying time or working with “wet” gloves (n = 9); however, 2 participants commented that ABHR did not take long to dry after it was applied to their gloves. Furthermore, 8 participants were concerned about undermining patient safety, particularly if HCP did not sufficiently sanitize the gloves or misused them (eg, using the same gloves when caring for multiple patients, or not replacing gloves when soiled). Also, 6 HCP were concerned that sanitizing gloves would take additional steps and time. In addition, 3 HCP were concerned about patient perceptions and experiences of care (eg, being touched with “wet” gloves), 2 HCP were concerned about reduced dexterity, and 1 HCP was concerned about compromised glove integrity after reuse.

**Table 4.** Healthcare Personnel Perceptions of Using ABHR to Cleanse Gloved Hands

Themes	Exemplar Quotes
Perceived benefits	• I guess it would be ok. I mean, it would be better than not changing gloves at all, you know. (Allied health professional, site 3, usual-care arm) • I don’t really see any drawbacks besides a little bit of extra time [compared to usual care], which would be worth if it was preventing infection… I think that it’s more reasonable [compared to ideal standard], in terms of the waste and the time. (Physician, site 3, intervention arm) • I think from a time efficiency point of view, it would be preferable. (Allied health professional, site 1, ideal standard arm) • I think that it would make things easier for folks… sanitizing and putting gloves over top of hands that have alcohol on them are not easy. (Physician, site 3, usual-care arm) • [It’s] just more protection, more cleaning, and more protection. (Physician, site 2, ideal standard arm) • There’s nothing sterile about them [gloves], they just sit around in a box all day. And, how many people have put their hands in that box? So, it makes sense. (Nurse, site 3, intervention arm) • I’d say it would save on glove cost. (Nurse, site 2, intervention arm)
Perceived concerns	• It [ABHR] took a while to absorb. It was a little bit annoying. (Allied health professional, site 1, intervention arm) • It’s a little odd, I feel like I can’t get it dry all the way. Like, I know it dries, but I feel like I can’t get it to dry, it feels wet. (Nursing assistant, site 2, intervention arm) • I really don’t think that doing that is actually going to kill all that bacteria on the glove. (Nursing assistant, site 3, usual care arm) • I guess they would need to make sure that the gloves they have won’t degrade down. (Physician, site 3, ideal standard arm) • Yeah, I think it would be harder [compared to usual care]. Just because you have to constantly go back and forth between the patient. And when you’re in there and, you know, have that motion, it can break the flow, the flow of your motion. (Allied health professional, site 3, intervention arm) • I don’t know, as a healthcare practitioner that puts my hands on the patients a lot, how much they would appreciate having slimy gloves. (Allied health professional, site 1, ideal standard arm)

## Discussion

In this 3-arm randomized trial performed within single patient encounters, we found that directly decontaminating gloved hands with ABHR may be a practical method for decreasing the bacterial load on gloved hands during patient care compared with usual care, which, as demonstrated in this study, is often characterized by poor adherence to gold standard practice. The following key findings of our study support this conclusion. First, compliance with the recommendations of the WHO Five Moments of Hand Hygiene (ie, the usual-care arm of study) was very poor and was associated with the highest percentage of gloves contaminated, highest total CFU count per glove, and highest number of pathogenic bacteria. Second, the intervention arm, decontaminating gloved hands with alcohol at each hand hygiene moment, was associated significant improvement in all the outcomes compared with the usual-care arm. The gold standard of removing gloves and performing hand hygiene was statistically superior to the intervention for all outcomes, but we and many other researchers^6,7,13,14,21^ have demonstrated that this practice is not feasible to implement, and compliance is poor in real-world settings. Directly decontaminating gloved hands with ABHR may be an optimal compromise to reduce contamination with an intervention that is more feasible and time efficient.

To our knowledge, our study is the first randomized trial to investigate the intervention of applying ABHR to gloved hands for the WHO Five Moments of Hand Hygiene within a given patient interaction. Our study compared usual care (what happens in practice) with the intervention. We also compared the intervention with the gold-standard WHO recommendation of removing gloves and performing hand hygiene at each moment, with this gold-standard practice enforced by trained observers.

The World Health Organization (WHO) 2009 guidelines recommend using ABHR or washing hands with soap and water during each of the Five Moments of Hand Hygiene: (1) before touching a patient; (2) before a clean or aseptic procedure; (3) after body fluid exposure risk; (4) after touching a patient; and (5) after touching patient surroundings. Other studies have found poor adherence to these recommendations and have shown that these recommendations are very time consuming.^6,7,13,14,21^ Numerous studies have shown that as the number of opportunities for hand hygiene increase hand hygiene rates decrease.^7^ Our findings revealed even poorer compliance with the Five Moments of Hand Hygiene compared to the literature. Prior studies cited were not completed in settings with mandatory glove use, such as our current study. Thus, this lower 2% compliance estimate highlights the impracticality of current gold-standard practice for glove wearers. Additionally, ABHR dispensers are not allowed at the bedside in the United States because of fire regulations, so that maintaining gold-standard practice may be even more difficult in the United States where this study was completed.

The strengths of our study include (1) randomization, (2) multicenter design, (3) mixed methods study design, and (4) outcome measurements, which that have been used by numerous other studies. Our study had several limitations. We measured the outcomes only at the end of the episode of care or when 7 hand hygiene moments were reached. Ideally, individual randomized trials would assess these outcomes at each hand hygiene moment since the contamination rates are not the same.^22–25^ In addition, we used an imprint plate method because it is less cumbersome and less time consuming for HCP participants than more complicated sampling methods such as the glove-juice method, and thus was more likely to be feasible. A criticism of the imprint plate method is that it does not sample the entire hand and thus may not yield identical results to the glove-juice method.

We do not know what the compliance with the proposed intervention arm would be if implemented in clinical settings. In the intervention arm, the study coordinator stopped the HCPs; thus, compliance could not be measured. Future studies should consider estimating compliance with the intervention arm in clinical (ie, nontrial) settings in a blinded fashion. The microperforation rate in the intervention arm was higher than the rate in the usual-care arm. These results are concerning and should lead to future work assessing these findings in more depth and possible novel technological discoveries to improve gloves to prevent microperforation due to alcohol-based decontamination.

In conclusion, given the benefits of this intervention, the CDC and the WHO and other health organizations should consider advising healthcare personnel to decontaminate their gloved hands with alcohol when hand hygiene moments arise during single patient encounters. This recommendation would not change the goal of achieving nearly 100% adherence with glove removal and proper hand hygiene upon patient room exit, and it could lower the risk of HCP transmitting pathogens to clean body sites and indwelling devices without hampering HCP’s ability to perform multiple patient care tasks during a single patient encounter.
